# Glutathione–Hemin/Hematin Adduct Formation to Disintegrate Cytotoxic Oxidant Hemin/Hematin in Human K562 Cells and Red Blood Cells’ Hemolysates: Impact of Glutathione on the Hemolytic Disorders and Homeostasis

**DOI:** 10.3390/antiox11101959

**Published:** 2022-09-30

**Authors:** Sofia K. Georgiou-Siafis, Martina K. Samiotaki, Vassilis J. Demopoulos, George Panayotou, Asterios S. Tsiftsoglou

**Affiliations:** 1 Laboratory of Pharmacology, Department of Pharmaceutical Sciences, School of Health Sciences, Aristotle University of Thessaloniki (AUTh), 54124 Thessaloniki, Greece; 2 B.S.R.C. “Alexander Fleming” Vari, 16672 Attiki, Greece; 3 Laboratory of Pharmaceutical Chemistry, Department of Pharmaceutical Sciences, School of Health Sciences, Aristotle University of Thessaloniki (AUTh), 54124 Thessaloniki, Greece

**Keywords:** GSH, hemin/hematin, K562 cells, RBC hemolysates, intracellular level of hemin, GSH–hemin adducts, GSH–hematin adducts, *N*-acetyl cysteine, metabolites, hemolytic disorders

## Abstract

Hemin, an oxidized form of heme, acts as potent oxidant to regulate glutathione (GSH) content in pro-erythroid K562 nucleated cells, via activation of the KEAP1/NRF2 defensive signaling pathway. Moreover, GSH, as an essential metabolite, is involved in the regulation of cell-redox homeostasis and proposed to scavenge cytotoxic free heme, which is released from hemoglobin of damaged red blood cells (RBCs) during different hemolytic disorders. In the present study, we aimed to uncover the molecular mechanism by which GSH inhibits hemin-induced cytotoxicity (HIC) by affecting hemin’s structural integrity in K562 cells and in RBC hemolysates. GSH, along with other thiols (cysteine, thioglycolic acid, and mercaptoethanol) altered the spectrum of hemin, while each of them co-added with hemin in cultures of K562 cells prevented HIC and growth arrest and markedly reduced the intracellular level of hemin. In addition, GSH endogenous levels served as a barrier to HIC in K562 cells, as shown by the depletion in GSH. LC-MS/MS analysis of the in vitro reaction between hemin and GSH revealed at least five different isomers of GSH–hemin adducts, as well as hydroxy derivatives as reaction products, which are characterized by unique mass spectra (MS). The latter allowed the detection of adducts in human RBC hemolysates. Based on these findings, we proposed a molecular mechanism via which GSH prevents HIC and structurally disintegrates heme. An analogous reaction was observed in RBC hemolysates via direct inter-reaction between hematin (ferric and hydroxide heme) released from hemoglobin and GSH. Overall, GSH–hematin adducts could be considered as novel entities of the human metabolome of RBCs in hemolytic disorders.

## 1. Introduction

Heme (iron (II)-protoporphyrin IX) is the pigment of life in animals, serving as the essential carrier of oxygen in blood [1,2]. Red blood cells (RBCs) contain a large amount of heme bound in hemoglobins, synthesized de novo daily. During erythropoieisis, a tight regulation between the biosynthesis of heme and globins exists to avoid the accumulation of unbound free heme [3]. Equally important to homeostasis is the targeted catabolism of heme. Aged RBCs that fail to pass through the narrow sinus wall of the reticuloendothelial system are subject to phagocytosis, where heme is enzymatically degraded by heme oxygenase-1 (HO-1) [4]. Moreover, eryptotic RBCs are marked with specific signals, such as phosphatidylserine, to be recognized by macrophages during blood circulation [5]. All of these homeostatic mechanisms are mandatory, because heme possesses a Janus-face attitude becoming a hazard (toxic) when it is not bound to hemoglobin [6]. Hemolytic disorders are classified as intravascular disorders (i.e., hemoglobinopathies such as sickle cell disease, thalassemia, sepsis, malaria, and other conditions) or extravascular ones (i.e., hemorrhagic strokes and traumas) [7,8,9]. Under these conditions, heme formed extracellularly, by auto-oxidative reactions from hemoglobin [10], is free to act as a potent oxidant and a pro-inflammatory molecule [11]. Vascular occlusion, damage of the endothelium, kidneys, and liver, and secondary brain damage are all complications of hemolysis associated with free heme toxicity [12,13,14,15].

Due to the innate cytotoxicity of free heme, RBCs *per se* are equipped during their lifespan with mechanisms to halt the autoxidation and release of heme from hemoglobin. When hemoglobin delivers oxygen to tissues, there is a risk of hemoglobin being converted to methemoglobin, which is unable to carry oxygen and prone to loose hematin (the oxidized form of heme) [16]. Moreover, RBCs are continuously exposed to oxidative stress, as occurs in the oxygen-rich environment of the lungs. RBCs are equipped with a complicated antioxidant system, consisting of both enzymatic and non-enzymatic constituents. Special attention is paid to the reduction of methemoglobin by the reductase of methemoglobin, as well as by GSH and ascorbic acid. GSH, as an important metabolite, is at the center of the antioxidant system of RBCs; it is found at concentrations of approximately 1 mM to 2 mM per RBC [17,18,19,20].

GSH, a tripeptide of γ-glutamyl-cysteinyl-glycine, is a powerful antioxidant involved in the scavenging of reactive oxygen species (ROS), directly or through the action of GSH peroxidase [21,22]. Moreover, the detoxification of a myriad of xenobiotics involves their conjugation to GSH by GSH transferases [23]. GSH oxidized to the glutathione disulfide (GSSG) is recycled back by GSH reductase. Importantly, the pathways for both the biosynthesis and the metabolism of GSH are highly active in RBCs [16,24]. Over the years, data have shown that GSH can be the cytosolic constituent of RBCs that directly scavenges heme released from hemoglobin under various pathophysiological conditions. A molar excess of GSH protects from hemin (the oxidized form of heme, used at in vitro induced hemolysis [25,26]). Via spectrophotometric analysis, GSH has been shown to react with hemin, leading to the release of iron [26,27,28]. Heme degradation by GSH has been proposed to account for the high levels of non-heme iron found in the membranes of hemoglobinopathic RBCs [28].

In our previous work, we observed that hemin (at the sub-cytotoxic concentrations of 20 μΜ to 50 μΜ) activates the KEAP1-NRF2 stress-response signaling pathway in K562 pro-erythroid cells, leading to the transcriptional activation of the NRF2-target genes, the glutamate cysteine ligase catalytic subunit (*GCLC*), and the cystine-glutamate antiporter (*xCT*), and to an increase in the biosynthesis of GSH [29]. The thresholds in hemin intracellular levels determine the progression of hemin-induced cytotoxicity (HIC) from cell cycle arrest to cell death. At the same time, proteomic analysis has shown that hemin intracellularly binds with high affinity to various heme-binding protein species, which are part of versatile signaling pathways [30]. Interestingly, the prototype thiol, *N*-acetyl-cysteine (NAC), when added in culture in excess to hemin (0.5 mM to 5 mM), prevents HIC via keeping the intracellular levels of hemin manageable [31]. The chemical reaction between NAC and hemin leads to the structural deterioration of hemin via the formation of covalent NAC–hemin adducts, as recently identified [31].

In the present study, we aimed to (a) decipher the prominent role of endogenous GSH in the HIC progression in K562 cells depleted in GSH, by incubating them with buthionine sulfoximine (BSO), an inhibitor of the glutamate cysteine ligase (GCL) enzyme; (b) identify the biochemical mechanism via which the exogenous GSH added in excess to hemin rescues K562 cells from HIC, by measuring GSH, GSSG, and hemin intracellular levels, as well as cell growth and death, in the presence and absence of hemin, GSH, and/or BSO; (c) delineate the chemical mechanism of reaction between GSH and hemin by employing LC-MS analysis in the in vitro reaction products; and (d) search and identify the newly characterized reaction products that are formed between GSH and hemin/hematin in healthy human RBCs, and their experimental hemolysates, based on fragmentation mass spectra (MS). If successful, this work can provide experimental evidence that GSH reacts with and deteriorates heme derived from hemoglobin, yielding novel metabolites that affect homeostasis and disease.

## 2. Materials and Methods

### 2.1. Cell Culture and Reagents

K562 cells (ATCC and CCL-243^TM^) were cultured in RPMI-1640 medium [32]. Hemin (Sigma-Aldrich, St. Louis, MO, USA) was prepared in an alkaline solution [33]. The fetal bovine serum (FBS), antibiotics-antimycotics (Gibco, Thermo Fisher Scientific, Waltham, MA, USA), BSO (Sigma-Aldrich, St Louis, MO, USA), N-acetyl cysteine (NAC), and GSH used in the cell culture experiments were >97% pure.

### 2.2. Assessment of Cell Growth and Viability

K562 cells were counted in a Neubauer hemocytometer, while cell viability was determined by the trypan blue (Sigma-Aldrich, St Louis, MO, USA) exclusion assay [34].

### 2.3. Determination of GSH and GSSG Levels

The enzymatic recycling method was employed to determine the GSH equivalents (GSH + 2GSSG) and the glutathione disulfide (GSSG) levels [35]. Standard curves of GSH and GSSG facilitated the quantitative analysis. A kinetic approach was conducted for both the samples and the standards, determining the absorbance (at 405 nm) at different times of reaction (0 min to 15 min). The kinetic results (absorbance at 405/min) were plotted to calculate the slopes for both the samples and the standards. The slope of each concentration of GSH or GSSG was re-plotted against the concentration employed, yielding the standard curves. The results were normalized as nanomoles (nmoles) of GSH equivalents and nmoles of GSSG levels/10^6^ cells. 5,5′-dithiobis-(2-nitrobenzoic acid) (DTNB), glutathione reductase, GSSG, sulfosalicylic acid, triethanolamine, and 2-vinyl-pyridine were products of Sigma-Aldrich (St. Louis, MO, USA) and β-NADPH was a product of Panreac-Applichem (Barcelona, Spain).

### 2.4. Spectrophotometric Analysis of Hemin

The colorless RPMI-1640 culture medium (RPMI-11835, Gibco, Thermo Fisher Scientific, Waltham, MA, USA), supplemented as described previously [31], was used for the incubation of hemin (Sigma-Aldrich, St. Louis, MO, USA) with GSH, cysteine, mercaptoethanol, alanine (Sigma-Aldrich, St. Louis, MO, USA), or thioglycolic acid (a kind gift of Professor K. Tsoleridis, Department of Chemistry, Aristotle University of Thessaloniki). The spectrophotometric changes in hemin were recorded via a UV-visibility (UV/Vis) spectrophotometer (U-2000, Hitachi).

### 2.5. Quantitation of the Intracellular Hemin Level

A pyridine hemochromogen assay [36,37] was employed to determine the quantity of structurally intact hemin piled into K562 cells. The differences in absorbance (555 nm and 540 nm) of the spectrum recorded (U-2000, Hitachi) were used to quantitate hemin content. A standard curve of hemin facilitated the analysis, while the data were presented as nmoles of hemin/10^6^ cells.

### 2.6. Electrospray (ESI)-LC-MS/MS Analysis

#### 2.6.1. In Vitro Reaction

A 0.1 Μ sodium phosphate (pH 7.0) buffer was used as the reaction buffer for the incubation of hemin (50 μΜ), with excess GSH (2 mM) at 37 °C, for 2 h.

#### 2.6.2. LC-MS/MS Analysis

The HPLC and MS/MS analysis parameters were thoroughly described previously [31,38]. The LTQ Orbitrap XL mass spectrometer (Thermo Fisher Scientific, Waltham, MA, USA) was employed throughout this study.

#### 2.6.3. Quantitation of molecular Ions

The molecular ion peaks corresponding to hemin and hematin (616.18 *m*/*z*) and the reaction products between GSH and hemin/hematin (at 461.12 *m*/*z*, 921.23 *m*/*z*, and 470.13 *m*/*z*) were quantified automatically in a Thermo Xcalibur Qual Browser (genesis, area under the curve).

### 2.7. Isolation of RBCs

Whole blood (EDTA anti-coagulated, derived from a healthy volunteer) was centrifuged (500× *g*, 5 min). The buffy coat was carefully discarded and the pellet of RBCs was washed three times (PBS).

### 2.8. Extraction of Cellular Low Molecular Weight (LMW) Metabolites

K562 cells and/or RBCs were suspended in 50% acetonitrile (pre-chilled, added three times over the volume of pellets) to remain on ice (10 min). This served to quench the “self-reaction” of hematin with GSH during the extraction procedure. The extracts were thoroughly vortexed and centrifuged (10.000× *g*, 10 min, 4 °C). The supernatant was analyzed by LC-MS/MS [modified by [39]].

### 2.9. Statistical Analysis

The mean ± S.D. from three (n) independent experiments was applied in each experiment. Pairwise t-test analysis was employed for the statistical analysis, where *p* values below 0.05 (*), 0.01 (**), and 0.001 (#) were assessed as statistically significant.

## 3. Results

### 3.1. Endogenous GSH Served as a Defensive Barrier to HIC in Default Cell Culture Conditions in K562 Cells

**Figure 1:**
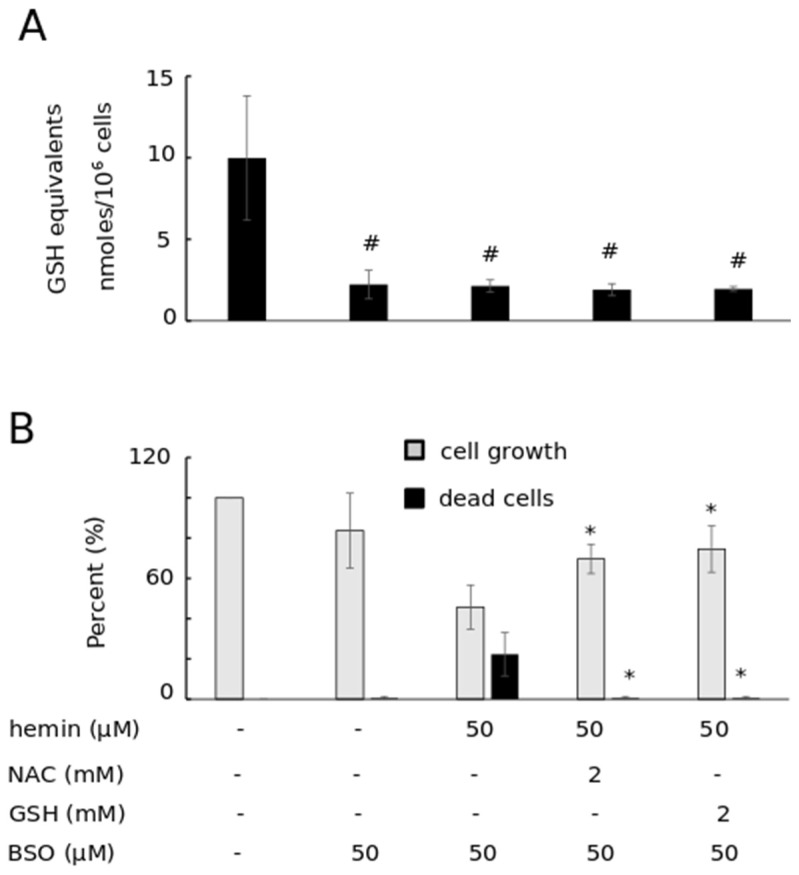
Total GSH equivalent levels, cell growth, and viability in K562 cells treated with the chemical inhibitor of GSH biosynthesis (BSO), in the presence of hemin and/or NAC and/or GSH. Κ562 cells were pre-incubated with BSO (50 μΜ) for 24 h. Thereafter, cells were washed out and re-suspended in cell medium without any further addition or with hemin (50 μΜ), or with hemin in presence of NAC and/or GSH (2 mM), and incubated for another 24 h. Afterwards, the quantity of GSH equivalents (**A**), cell growth, and viability (**B**) were determined. Cell growth percentage was calculated relative to K562 cells treated with no agents from the onset of the experiment (cell growth at 100%) and expressed as a percentage. Cell viability was determined as the percentage of trypan blue positive cells in each culture. Statistical differences were shown between cells pre-incubated with BSO and those left untreated (**A**) or cells treated with hemin and BSO and those treated simultaneously with either NAC or GSH (**B**). *p* values < 0.05(*) and 0.001 (#).

As mentioned in the Introduction, K562 pro-erythroid cells respond to HIC through the targeted induction of GSH biosynthesis-related genes [29]. The first question we asked related to the contribution of endogenous GSH in the cellular defense of K562 cells from HIC. We followed a biochemical approach involving the inhibition of the biosynthesis of GSH using BSO, an irreversible, competitive inhibitor of GCL holoenzyme. The pre-incubation of cells with BSO at 50 μΜ for 24 h, based on previous study [40], resulted in a significant reduction of GSH (by ~80%,compared with the GSH levels of unblocked cells) (Figure 1A). Interestingly, the depletion in intracellular GSH content to these levels was found to persist even 24 h after the removal of BSO from the media of the cells (Figure 1A). Several types of cells cannot easily regenerate the GSH pool, because BSO is not readily catabolized intracellularly [41]. 

Moreover, cell growth was moderately effected in cells pre-treated with BSO and then washed out (at 80% of the untreated cells), while cell death was not recorded (Figure 1B). Under these conditions, a cellular model to study the cytotoxic effects of hemin in cells with markedly reduced/depleted levels of GSH was established. Upon the removal of BSO, hemin was added and both GSH levels, as well as cell growth and viability, were assessed 24 h thereafter. Hemin did not increase the GSH content in the BSO-blocked cells (Figure 1A, BSO/hemin-treated cells), as expected based on the chemical inhibition of the GCL holoenzyme. It was found that the depletion of GSH significantly worsened HIC, as demonstrated by the proportion of dead cells, which was determined to be 22% (Figure 1B, BSO/hemin-treated cells), in contrast to viable cells that remained in culture treated with hemin-only for the same time period (24 h) [29]. These data indicated that endogenous GSH, induced by hemin biosynthetically, served as defensive barrier to HIC.

### 3.2. The Exogenous Addition of GSH Rescued K562 Cells from HIC: Analysis of the Molecular Mechanism

As found above, endogenous GSH served to halt the progression of HIC in K562 cells from cell cycle arrest to cell death. GSH or NAC, when added in excess to hemin in culture, rescues RBCs from hemin-induced hemolysis [25,27] or K562 cells, neurons, and other cell types from HIC [31,42]. To identify the molecular mechanism of protection underpinning this process, we explored two different scenarios, which are presented below.

#### 3.2.1. GSH Biosynthesis Increased in Thiols (NAC and GSH)-Induced Prevention of HIC

**Figure 2:**
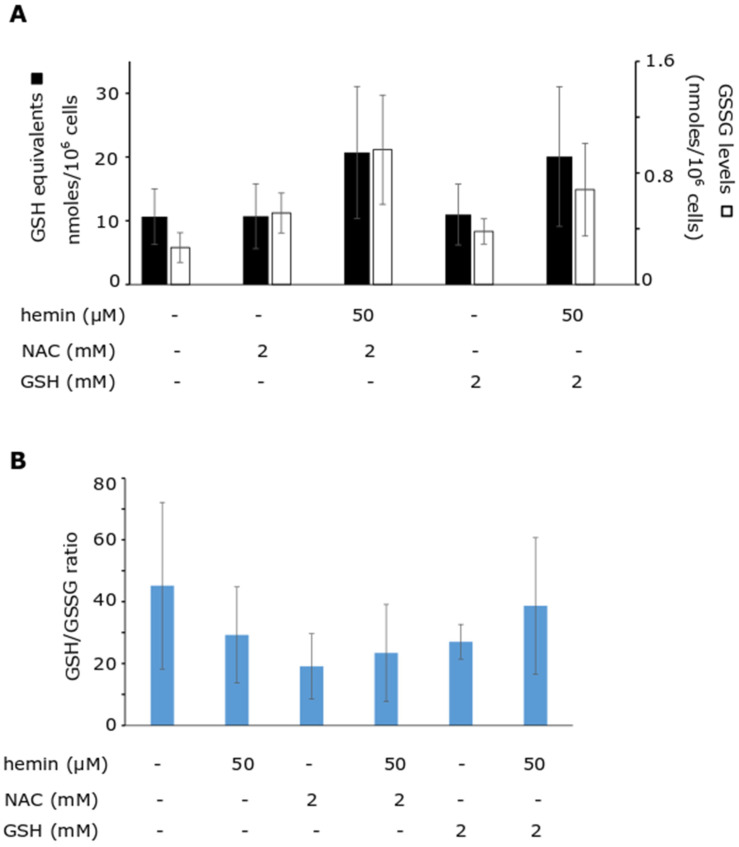
GSH equivalents, GSSG levels, and GSH/GSSG ratio in K562 cells treated exogenously with GSH or NAC in the presence and absence of hemin. (**A**) K562 cells were exposed to either NAC or GSH (2 mM), in the presence and absence of hemin (50 μM) for 12 h. Thereafter, GSH equivalents and GSSG levels were determined. Cells without any addition served the as the experiment controls. (**B**) The ratio of GSH/GSSG was calculated by dividing the GSH (reduced form) level (calculated by subtracting the GSSG levels (2×) from GSH equivalents) by the GSSG (oxidized form) level of each sample shown in Figure 2A.

The first scenario was carried out to provethat GSH rescues K562 cells from HIC through the replenishment of the endogenous GSH pool. To investigate this possibility, we determined the intracellular pool of GSH in K562 cells treated exogenously with a molar excess of GSH, in the absence and presence of hemin. NAC, as a donator of cysteine, was used as a known substrate for GSH biosynthesis [43,44]. Neither NAC nor GSH alone increased the cellular GSH equivalents, depicting a negative regulatory loop (Figure 2). Either NAC or GSH, co-added to cells with hemin, increased (doubled) the GSH equivalents over those results observed in cells treated with either thiol alone or with hemin alone (GSH equivalents at 16.99 ± 3.27 nmoles/10^6^ cells, GSSG levels at 0.61 ± 0.22), as published recently by our research group [29,31]. GSH and NAC were able to increase the GSH intracellular content only under the condition of increased GSH biosynthesis attributed to hemin-induced stress. An increase in GSSG was detected, especially in the case of NAC addition. NAC is known to be a weak antioxidant itself [45,46]. However, the largest part of the GSH pool remained in its reduced form (Figure 2). Moreover, the ratio of GSH/GSSG decreased in cells that were treated with either hemin or NAC alone and/or with hemin and NAC, implying an impaired redox status. In the case of exogenous GSH addition, the ratio of GSH/GSSG decreased less prominently, compared with the ratio for untreated cells.

#### 3.2.2. GSH Biosynthesis Was Not a Key Mediator of Thiols (NAC and GSH)-Induced Prevention of HIC

To determine the role of endogenous GSH in the thiols-induced prevention of HIC, we returned to our cellular model of GSH pool reduction (Figure 1). When NAC or GSH was added simultaneously with hemin to the BSO-blocked cells, the GSH levels were not increased (Figure 1A, BSO/hemin/NAC- or BSO/hemin/GSH-treated cells). This result is readily explained for NAC, because it is used as a precursor for GSH, either by itself after intracellular deacetylation or through the extracellular reduction of cystine, the predominant form of thiols in extracellular fluids [43,44]. Accordingly, under chemically (BSO) inhibited GCL holoenzyme conditions, NAC could not be used for GSH biosynthesis. However, an increase in GSH levels was not detected even by the exogenous addition of GSH, which indicated undoubtedly that exogenous GSH was not used intact by K562 cells. GSH does not easily enter the cells, but degrades through the action of membranous γ-glutamyl transpeptidase to glutamic acid, cysteine, and glycine, all serving as the real substrates for GSH biosynthesis [47].

In addition to the significantly diminished GSH pools, both NAC and GSH treatment protected cells to a remarkable degree from HIC (Figure 1B). Cell death associated with BSO/hemin-treated cells was reduced from 22% to 0%, while cell growth was increased from 45% to 70% and 75% in the cases of BSO/hemin/NAC- and BSO/hemin/GSH-treated cells, respectively (Figure 1B). Even during the significant reduction in endogenous GSH, thiols, added exogenously, protected from HIC. NAC accomplished this in a way that was comparable to that of GSH. We should emphasize that BSO-treated cells grow up to 80% of untreated cells.

#### 3.2.3. Management of HIC, a Common Property of Thiols, Involved Hemin Structural Destabilization and Inhibition in Intracellular Accumulation of Hemin

**Figure 3:**
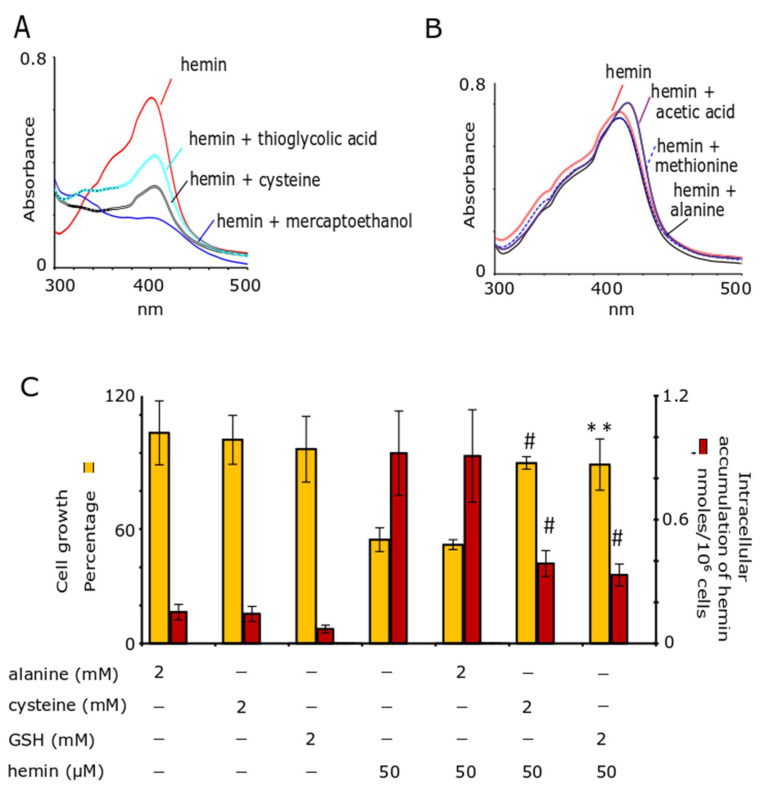
The effect of different thiols (GSH, cysteine, thioglycolic acid, and mercaptoethanol) on the spectrum of hemin, as well as of GSH and cysteine, on cell growth and the intracellular content in hemin. (**A**,**B**) Hemin (50 μΜ) was incubated with each one of the thiols and/or with alanine or acetic acid or methionine (2 mM) in a cell culture medium at 37 °C for 12 h. Spectrophotometric changes of hemin were then recorded. The spectrum of hemin in the presence of GSH is presented in the Supplementary Materials [31] and is also presented here, for reasons of clarity. (**C**) K562 cells were incubated simultaneously with hemin (50 μΜ) and with alanine, cysteine, and/or GSH (2 mM) and then the intracellular content of cells with hemin (at 12 h) and cell growth (at 24 h) were determined. Cell growth is presented relative to the growth of untreated cells (100%). Cells incubated with only hemin and/or alanine, cysteine, or GSH served as the experimental controls. Statistical differences are shown between cells treated with hemin only and those treated simultaneously with hemin and GSH or cysteine. *p* values < 0.01 (**) and 0.001 (#).

Based on the above data, we explored the second scenario involving GSH being in reaction with hemin, as soon as their extracellular co-addition. When hemin is incubated with GSH in cell culture medium, a significant decrease in the spectrum of hemin was recorded, which is characteristic of its protoporphyring ring (Figure 3A). Other thiols (cysteine, mercaptoethanol, and thioglycolic acid), when tested in the same way, also decreased the spectrum of hemin (Figure 3A). The spectrum of hemin almost disappeared when hemin co-incubated with mercaptoethanol. In contrast, agents that either lack the free sulfhydryl group (–SH) (such as alanine as an analog to cysteine and acetic acid as an analog to thioglycolic acid), or that have the GSH substituted (methionine), did not affect its spectrum (Figure 3B).

Cells were then co-exposed simultaneously to both hemin and each of the cysteine or GSH (i.e., the natural-origin ones). Cells that were co-exposed to both hemin and alanine, an amino acid without free GSH, served as the experiment controls. Both cysteine and GSH, unlike alanine, reversed completely hemin-induced growth arrest and attenuated hemin cellular content to the level (at ~0.3 nmoles/10^6^ cells, see Figure 3C) recorded in our previous studies as being manageable by cellular detoxification mechanisms, such as HO-1 [29,31]. Therefore, thiols in general and GSH in particular exhibited the capacity to attenuate the intracellular accumulation of hemin to rescue cells from HIC.

In summary, we followed two apparently opposite approaches, either inhibiting GSH biosynthesis or adding GSH in excess in cell culture medium, in the frame of HIC. From these sets of data, we reached the following conclusions: (a) in default cell culture conditions, GSH endogenous levels, induced by hemin, protected K562 cells from HIC aggravation; and (b) upon the extracellular addition of GSH in excess, its specific reaction with hemin was the key event in the successful prevention of HIC. The molecular basis of protection by GSH involved the maintenance of an intracellular safe level of hemin. In this context, the replenishment of the intracellular GSH pool was not a prerequisite to prevent HIC. In either case, GSH proved to be an important player in restriction of HIC.

### 3.3. Novel Covalent Adducts between GSH and Hemin (GSH–Hemin Adducts) and Their Derivatives Were Identified in the In Vitro Reaction between GSH and Hemin

**Figure 4:**
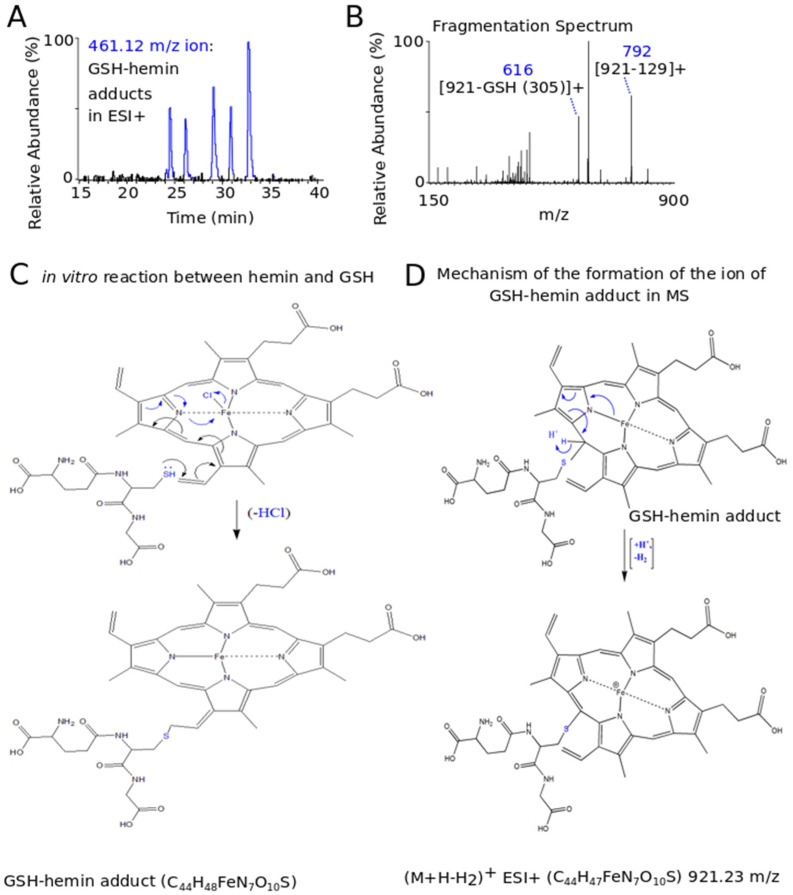
GSH–hemin adducts were uncovered as the products in the in vitro reaction between GSH and hemin. Hemin (50 μΜ) was incubated with GSH (2 mM) at 37 °C for 120 min. Thereafter, the reaction products were full-spectrum (200–2000) *m*/*z* analyzed in LC-MS, and the gained data files were assessed. The ion chromatograph (**A**) and the fragmentation spectrum (**B**) of the 461.12 *m*/*z* ion, representing the divalent molecular ion of GSH–hemin adduct. (**C**) The mechanism of reaction between GSH and hemin yielding GSH–hemin adducts. One isomer is presented, where the addition of GSH took place at the vinyl group (side chain). (**D**) The mechanism of the formation of the 921.23 *m*/*z* (monovalent ion of GSH–hemin adduct), detected in MS, is shown by the arrows. The isomer of GSH–hemin adducts at one of the four ethylene bridges of hemin is shown here. The ChemDraw Ultra 8.0. was used to design the chemical structures. *m*/*z*: mass-to-charge ratio, ESI+: positive electrospray.

To understand the chemical mechanism of the reaction between hemin and GSH, LC-MS analysis was performed. In each experiment, a hemin-only (control) experiment was carried out for consistency. Hemin (C_34_H_32_ClFeΝ_4_O_4_) (M) was represented as a molecular ion of 616.18 *m*/*z* (M-Cl)^+^, eluted at 35 min. At the reaction between hemin and GSH (C_10_H_17_N_3_O_6_S, 307.08 Da), a molecular ion at 921.23 *m*/*z*, as well as one at 461.12 *m*/*z*, were found to be eluted consistently at the same retention times (RTs). In particular, both ions eluted at five RTs, ranging from 24 min to 32 min (Figure 4A). The multiple RTs corresponded to isomers of the GSH–hemin adducts (C_44_H_48_FeN_7_O_10_S) (Μ1), which were characterized by reduced hydrophobicity, as eluted significantly earlier compared to hemin. The average fragmentation spectrum of the 461.12 *m*/*z* parental ion included as the daughter ion the 616 *m*/*z* (921–305 (GSH)) (Figure 4B). Furthermore, the 129-neutral loss, a molecular mark of the thioether bond [48], was found [daughter ion at 792 (921 (parent ion) minus 129 (neutral loss)].

The 921.23 *m*/*z* ion represented the (M1+H-H2)^+^ of the proposed product (C_44_H_48_FeN_7_O_10_S) (M1), derived by the nucleophilic addition of GSH to hemin. A thioether bond was formed resulting in electron rearrangement and the generation of an additional covalent bond between nitrogen and iron (Figure 4C). Finally, a molecule of HCl was subtracted, yielding novel derivatives of hemin (GSH–hemin adducts, M1) (Figure 4C). The structure of the (M1+H-H2)^+^ molecular ion and the mechanism leading to its detection in MS analysis is shown at Figure 4D. The 461.12 *m*/*z* ion [(M1+H-H2)+H]^2+^ was derived from protonation of the amine group of the GSH side chain. The divalent 461.12 *m*/*z* was found in the largest quantity in the in vitro reaction, rather than the 921.23 *m*/*z* ion, and the fragmentation spectrum (MS2) was, thus, received only for the former.

**Figure 5:**
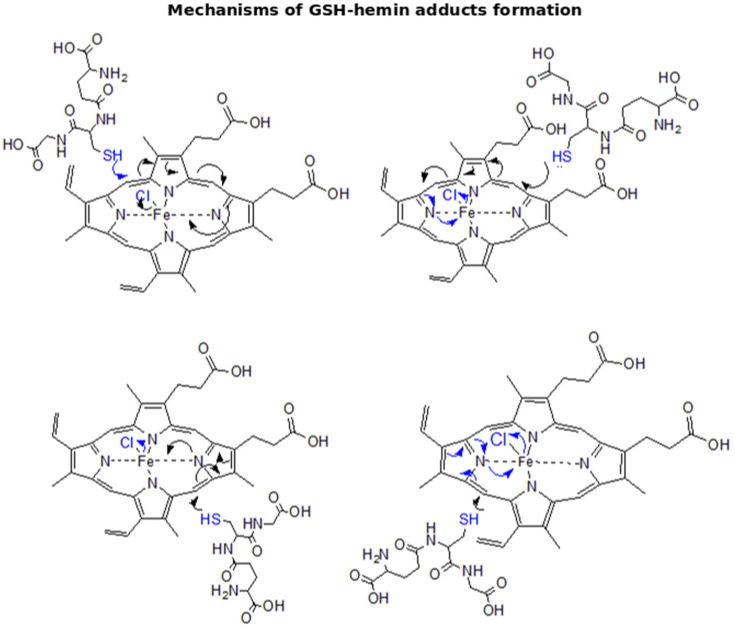
Proposed mechanisms of nucleophilic addition in the ethylene bridges of hemin by GSH yielding isomers of GSH–hemin adducts.

Multiple sites (ethylene bridges and side chains) (as shown at Figure 5) are at GSH disposal for the nucleophilic addition, as can be drawn by the existence of five isomers of the GSH–hemin adduct.

**Figure 6:**
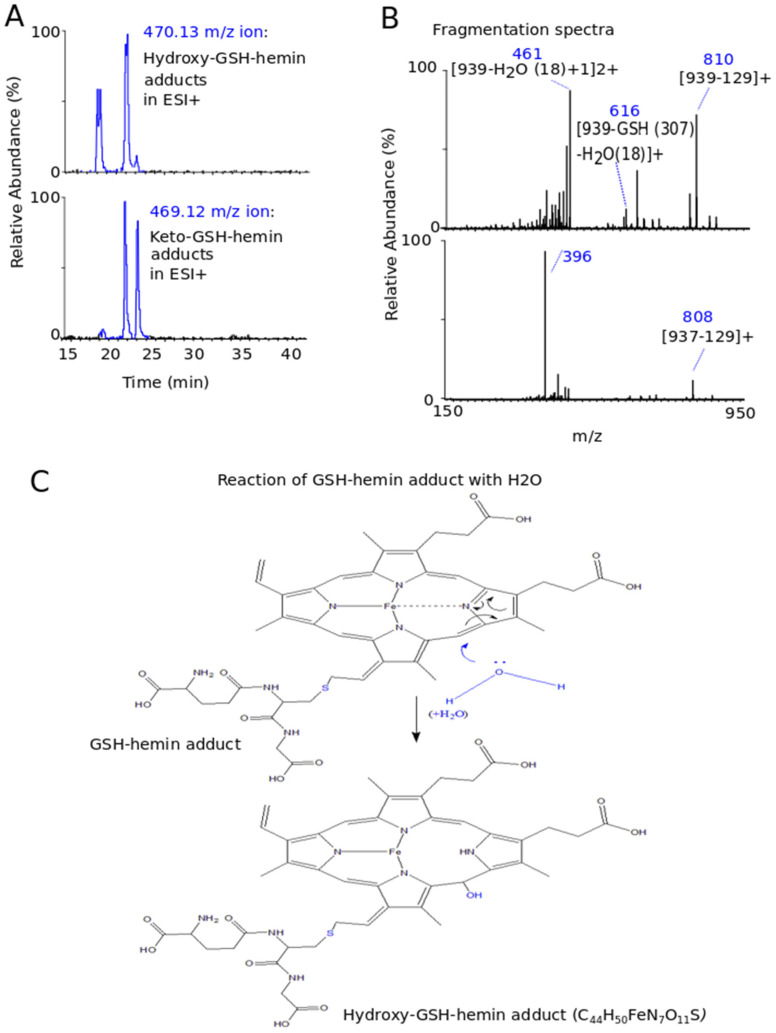
Formation of hydroxy–GSH–hemin adducts at the in vitro reaction between GSH and hemin. The ion chromatographs (**A**) and fragmentation spectra (**B**) of the 470.13 *m*/*z* and 469.12 *m*/*z* ions, corresponding to the [(M+H-H2)+H]^2+^ of hydroxy–GSH–hemin adducts and keto–GSH–hemin adducts, respectively. (**C**) The mechanism of reaction of GSH–hemin adducts with water and the resulting structure of the hydroxy–GSH–hemin adducts (one isomer) is shown.

Additional molecular ions at 939.24 *m*/*z* and 937.22 *m*/*z* were formed at the reaction between hemin and GSH. These ions preceded the elution of GSH–hemin adducts in LC-MS analysis (RTs between 18 min and 22 min) (Figure 6A) (Figure 4A). They are proposed as the (M+H-H2)+ of the hydroxy (C_44_H_50_FeN_7_O_11_S) (M2) and its keto derivatives (C_44_H_48_FeN_7_O_11_S) (M3) of the GSH–hemin adducts. As in the case of GSH–hemin adducts, the divalent molecular ions at 470.13 *m*/*z* and 469.12 *m*/*z* were more abundant than their respective monovalent ions. As shown at Figure 6C, a molecule of water added to the GSH–hemin adducts yielded a pyrrole ring and ruptured the coordination bond between nitrogen and iron. Keto–GSH–hemin adducts were formed from the hydroxy-GSH–hemin adducts by air oxidation. An analogous mechanism to this, shown at Figure 4D, is proposed for the formation of the 939.24 *m*/*z* and 937.22 *m*/*z* ions, detected in MS. MS2 spectra data (Figure 6B) were in agreement with the characterization of these molecular ions as products of GSH–hemin adducts.

The reaction of GSH with hemin yielded the newly characterized GSH–hemin adducts, as well as their hydroxy and keto derivatives. Based on these products, we were able to delineate the chemical mechanism of the reaction between GSH and hemin (Figure 4, Figure 5 and Figure 6). Moreover, the weakening of iron bonding to hydroxy–GSH–hemin and keto–GSH–hemin adducts can explain the loss of iron and structural deterioration of hemin, caused by GSH [31].

### 3.4. GSH–Hematin Adducts Were Identified in Traces at Healthy Human RBCs and Intensified at Experimental Hemolysates

At this point, we were able to exploit the newly identified (by MS technology) reaction products formed between GSH and hemin in order to investigate whether GSH reacted with free heme in situ (inside cells). K562, human, pro-erythroid cells treated with hemin were our first-choice cellular sample, as we already knew that endogenous GSH limited the severity of HIC (Figure 1A). Next, we moved to human RBCs, where both reactants (GSH and hematin) are in excess, while their inter-reaction has been widely accepted through spectrophotometric and physicochemical methods for more than 35 years [25,26,27,28].

An extraction protocol of cellular low molecular weight (LMW) metabolites, based on the organic phase to partition the protoporphyrins (hemin and its derivatives), was adapted from previous work [39]. This extraction protocol was preferred instead of filter-assisted extractions that (in our preliminary experiments) significantly attenuated the quantity of hemin. A possible explanation is that hemin, as a “sticky” molecule, binds non-specifically to cellular proteins [49]. In the LC-MS analysis of extracts prepared from hemin-treated K562 cells, hemin was detected as a component (Figure S1A) at high quantity. However, GSH–hemin adducts, sought as the molecular ions of the in vitro reaction between hemin and GSH (Figure 4A), were not found. The same results were obtained in K562 cells treated simultaneously with NAC and hemin.

**Figure 7:**
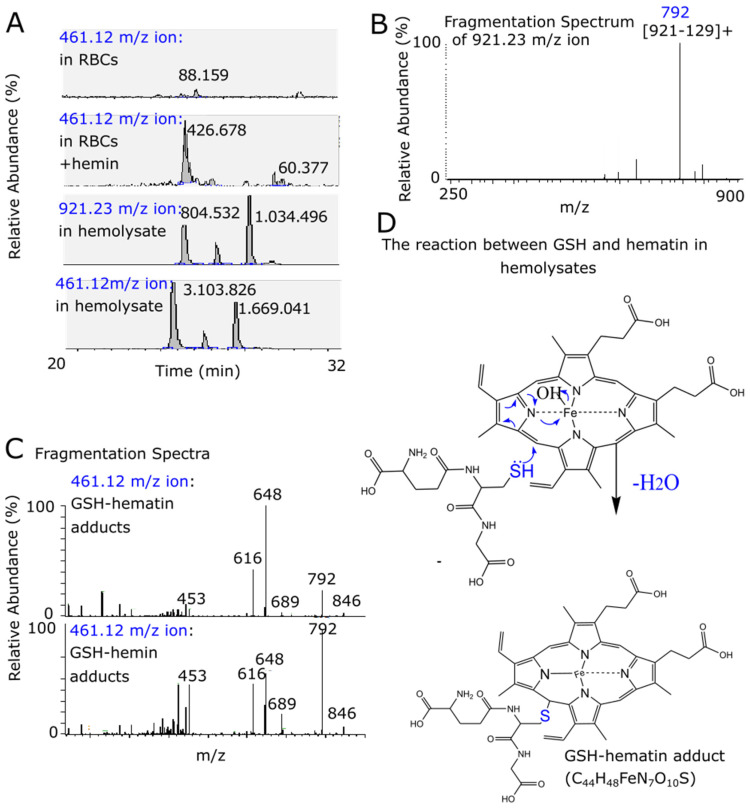
Identification of the 461.12 *m*/*z* and 921.23 *m*/*z* ions corresponding to GSH–hemin/hematin adducts in the hemolysates of human RBCs. (**A**) Ion spectra of the molecular ions detected at RBCs, as indicated. RBCs aliquots (~2 × 10^9^) were either proceeded with immediately, or incubated with 20 μΜ hemin (RPMI, 37 °C, 30 min) [these conditions were set to ”load” the RBCs with hemin, without hemolysis being provoked [28]] or lysed with water in the presence of acetonitrile (37 °C, 60 min) prior to the extraction. The quantification of the ion peaks is shown near each ion chromatograph. (**B**) The fragmentation spectrum of the 921.23 *m*/*z* ion, corresponding to (M1+H−H2)^+^ of the proposed GSH–hemin/hematin adduct (C_44_H_48_FeN_7_O_10_S). (**C**) Paired comparison of MS2 spectra of the 461.12 *m*/*z* ion detected at hemolysates and found in the in vitro reaction between GSH and hemin. (**D**) The mechanism of GSH–hematin adducts formation (one isomer is shown).

Having this in mind, we explored this question further. Healthy RBCs have been recently found to contain hematin (ferric hydroxide heme) (C_34_H_32_OHFeΝ_4_O_4_) (M’) derived from autoxidized hemoglobin, with a medium concentration among individuals at 20 μΜ [50]. In the LC-MS analysis of extracts derived from RBCs that were isolated immediately after their purification from whole blood, hematin was detected in high quantities, as the same molecular ion with hemin, at 616.18 *m*/*z* (M’-OH)+ (Figure S1C). Interestingly, a trace of the 461.12 *m*/*z* ion, which corresponded on the divalent ion of GSH–hemin adducts, was found (Figure 7A). Its intensity increased in RBCs exposed to hemin (Figure 7A). Due to its low quantity, the MS2 spectrum was not used to perform a paired comparison with the respective spectrum of GSH–hemin adducts (Figure 4B).

To gain further insight into the formation of the novel GSH–hematin adducts, we increased the quantity of hematin available for the inter-reaction with GSH. The RBCs were hypotonically lysed, and the resulting hemolysate was incubated for 1h at 37 °C in the presence of a low quantity of acetonitrile (10%). Biochemical studies concerning the denaturation/refolding of hemoglobin have proven that acetonitrile drives hemoglobin denaturation and hematin release [51]. In this case, the quantity of GSH–hematin adducts was significantly intensified (LC spectra intensity of 921.23 *m*/*z* and 461.12 *m*/*z* ions at >10^6^ relative abundance) (Figure 7A). The 921.23 *m*/*z* and 461.12 *m*/*z* ions represented the single- and double-charged ions of the GSH–hemin/hematin adducts. The single-charged ion was barely detectable in the in vitro reactions, due to the lower quantity that was formed. The MS2 spectrum of the 921.23 *m*/*z* ion is also shown in Figure 7B.

A paired comparison of MS2 spectra between the 461.12 *m*/*z* ion received in the in vitro reaction of GSH and hemin and the 461.12 *m*/*z* ion of the experimental hemolysate yielded (via the Thermo Xcalibur Qual Browser) high matching factors (SI is the direct matching factor; RSI is the reverse matching factor at >800) (Figure 7C). The nucleophilic reaction between hematin and GSH, as recorded in the in vitro reaction between hemin and GSH, yielded the same products in RBCs hemolysates (Figure 7D). In particular, the GSH–hemin adducts and the GSH–hematin adducts had the same chemical structure. The nucleophilic addition of GSH to hematin formed GSH–hematin adducts, while a molecule of water was subtracted.

**Figure 8:**
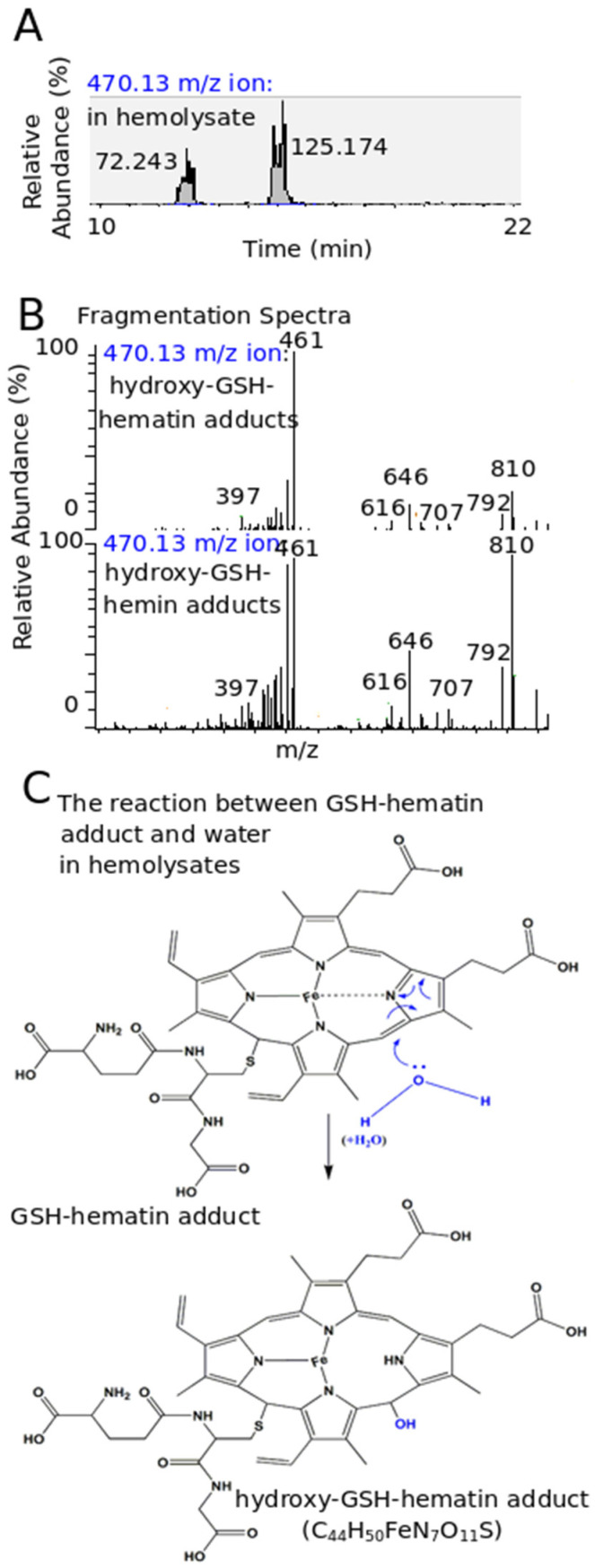
Identification of the 470.13 *m*/*z* ion corresponding to the hydroxy–GSH–hemin/hematin adducts in hemolysates of human RBCs. (**A**) Ion spectra of the 470.13 *m*/*z* ion, corresponding to hydroxy-GSH–hemin/hematin adducts at hemolysates (prepared as described at Figure 7A). The quantification of the ion peaks is also shown. (**B**) Paired comparison of MS2 spectra of the 470.13 *m*/*z* ion detected at the hemolysates and found in the in vitro reaction between GSH and hemin. (**C**) The mechanism of hydroxy–GSH–hematin adducts formation (one isomer is shown).

Moreover, the molecular ion at 470.13 *m*/*z* was identified as an abundant ion in the experimental hemolysate sample (Figure 8A). This mass was identical to that of divalent hydroxy–GSH–hemin adducts found in the in vitro reaction (Figure 6A,B). The 470.13 *m*/*z* ion represented the double-charged ions of the hydroxy–GSH–hemin/hematin adduct. A paired comparison between both 470.13 *m*/*z* ions showed high similarity scores (Figure 8B). The reaction of GSH–hematin adducts with water forming the hydroxy–GSH–hematin adducts is shown at Figure 8C. The detection of the hydroxy derivative indicated that a quantity of hematin was disintegrated by the GSH of RBCs during the 1 h incubation.

**Figure 9:**
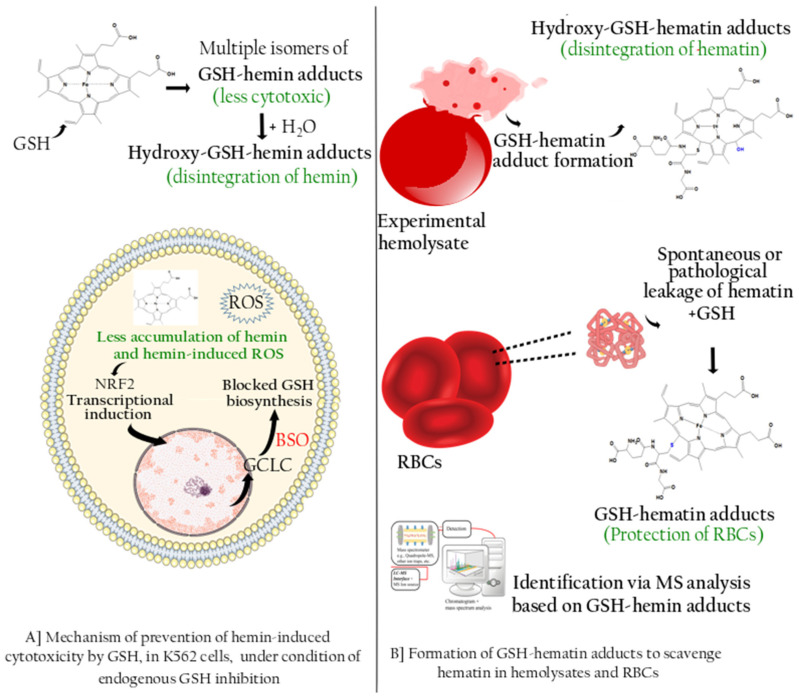
Illustration of the prominent role of GSH in managing HIC in K562 cells and RBCs. (**A**) GSH added in culture, in excess, reacts with hemin extracellularly forming GSH–hemin adducts. The latter are interconverted to hydroxy–GSH–hemin adducts, deteriorating hemin and releasing its iron. In this way, hemin intracellular accumulation is decreased, along with its cytotoxic actions (production of reactive oxygen species (ROS), inhibition of cell growth, and induction of cell death). At the same time, K562 cells were pre-incubated with BSO, an inhibitor of GCLC, the key biosynthetic enzyme in GSH biosynthesis. GCLC was induced by the action of hemin on the NRF2 transcription factor, but the GSH levels remained low due to the action of BSO. (**B**) (Upper panel) LC-MS analysis in extracts of hemolysates detected GSH–hematin adducts and their hydroxy derivatives that were formed in situ by hematin and GSH, both derived by RBCs. (Lower panel) GSH–hematin adducts were detected in traces of extracts prepared by intact, healthy human RBCs. Based on the important role of GSH, the putative role of GSH–hematin adducts in RBCs’ homeostasis is proposed. Parts of the figure were drawn using pictures from Servier Medical Art.

In this study, we provided experimental evidence showing that endogenous GSH leads to the generation of yet-unknown GSH–hemin/hematin adducts, formed spontaneously upon RBCs lysis in the presence of acetonitrile, accelerating hematin release. The nucleophilic reaction between hematin and GSH, as recorded in the in vitro reaction between hemin and GSH, yielded the same products in the RBCs’ extracts (Figure 8 and Figure 9). The biochemical events depicting GSH as an important metabolite in the management of HIC in pro-erythroid K562 cells, and RBCs are illustrated in Figure 9.

## 4. Discussion

### 4.1. GSH Was a Fundamental Constituent in the Management of HIC in K562 Cells: Analysis of the Mechanism

This work identifies a novel mechanism of interactions between the natural thiol (GSH) and cytotoxic (free) heme that is released from damaged RBCs in hemolytic disorders. HIC is a typical example of the disbalance of cellular redox homeostasis. Free heme intercalates into cellular membranes [52,53], where it provokes the production of ROS [54,55], protein, and lipid peroxidation [14,56]. GSH, on the other hand, is the most abundant low-molecular-weight thiol; it is an effective anti-oxidant of harmful ROS [21,23,57]. As recently found, hemin itself activates the antioxidant response pathway to counterbalance the oxidative damage of HIC to K562 cells [29]. The mechanism involves the inhibition of the KEAP1/NRF2 interaction by guiding KEAP1 for ubiquitination and subsequent degradation. The NRF2 transcription factor is free to translocate in the nucleus and induce its target-genes; among them are those related to GSH biosynthesis.

Here, two commonly applied experimental approaches were followed to investigate the GSH contribution to HIC at K562 cells. The first approach involved the chemical depletion in GSH levels and the second approach involved the supplementation with a molar excess of GSH (at mM concentrations). Hemin was added in both cases at a concentration (50 μΜ) known to provoke a time-dependent cytotoxicity, involving ROS production and cell-cycle arrest at 24 h of exposure, while cell death was induced later (at 48 h) [29]. In each approach, the role of GSH was prominent in the management of HIC. Its depletion made K562 cells more vulnerable to HIC, because cell death was accelerated and recorded at 24h (Figure 1). Hemin induces a cell death called feroptosis, an iron-dependent type of cell death involving ROS [58], while GSH depletion signals for glutathionylation as a critical regulator of apoptosis [22]. There have been several studies showing that endogenous GSH protects from several cytotoxic agents (such as chemotherapeutics) [41].

Here, endogenous GSH served as a barrier to HIC (Figure 1), probably via multiple mechanisms of action. First, GSH, as a potent antioxidant, can scavenge ROS produced intracellularly from the lipophilic hemin. Second, the involvement of the newly discovered reaction of GSH with hemin, leading to the formation GSH–hemin adducts and the decomposition of hemin (Figure 4, Figure 5 and Figure 6), should be investigated. However, hemin intracellularly does not easily remain free, as multiple hemin-binding proteins exist intracellularly [30,49]. Under our applied experimental conditions, GSH was able to react with hemin extracellularly, despite the presence of serum proteins (Figure 3A). In the first attempt to detect these GSH–hemin adducts intracellularly, no such products were detected (Section 3.4). The inability to detect them depended on several factors, such as the quantity of both the hemin and the GSH available intracellularly for the reaction, the structural instability of GSH–hemin adducts (as presented for NAC–hemin adducts [31]), and the number of K562 cells used in our analysis. As recorded in the literature, in well-documented GSH-oxidant reactions, such as in the formation of GSH–menadione adducts, those adducts are exported from renal tubular cells by multi-drug resistance proteins (MDRs) [59,60]. More studies on the cellular fate of the newly discovered GSH–hemin adducts need to be conducted. Third, the unambiguous role of endoplasmic reticulum GSH in disulfide formation [61] could aid hemin-stressed cells in encountering the oxidative stress, while proteasomal activity is not appropriate [62].

In most cells, the intracellular concentration of GSH is in the range of 1 mM to 2 mM, while in extracellular fluids it is much lower, as it is in the μM in human serum, whereas in the fluid of a thin layer of lung reaches mM concentrations [23,63]. When K562 cells were supplemented simultaneously with hemin and GSH, in molecular excess, HIC was prevented in both permissive GSH biosynthesis (Figure 3B) and chemically inhibiting conditions (Figure 1). This observation is controversial for the results analyzed above, where endogenous GSH was a key player in the restriction of HIC. However, this can be readily explained, as in presence of the extracellular molar excess of thiols (NAC or GSH), hemin intracellular levels did not reach cytotoxic levels (Figure 3B). GSH reacted with hemin as soon as their co-addition in the culture medium (Figure 3A) restricted the intracellular accumulation of hemin (Figure 3B). In the case of NAC, with respect to which a detailed study was previously conducted, the ROS accumulation, the NRF2-defense response, regarding as molecular signs of HIC, declined to minimum levels in K562 cells that were rescued from HIC by NAC [31]. Thiols administered as therapeutics, in cases such as acetaminophen poisoning and other disorders, act mechanistically by the replenishment of endogenous GSH levels [45]. In the case of GSH-induced protection from HIC, the real mechanism of protection was the quantitative formation of GSH–hemin adducts [64].

### 4.2. Elucidation of the Chemical Reaction between GSH and Hemin

By applying sophisticated LC-MS analysis, we detected and identified the reaction products formed by GSH and hemin, and we subsequently proposed the chemical mechanism of the reaction (Figure 4, Figure 5 and Figure 6). This reaction imposes an interconversion of GSH–hemin adducts to hydroxy–GSH–hemin and keto–GSH–hemin adducts. Neither product accumulated, as shown by a kinetic quantitative analysis performed for the in vitro reaction of NAC–hemin adducts [31]. Iron is released (detected by ferrozine analysis [25,26,27,28,31]) by the hydroxy–GSH–hemin and keto–GSH–hemin adducts (see [31] for the mechanism). These data led to our conclusion that the reaction between GSH and hemin proceeds through several intermediate labile products, detoxifying hemin, while the final product(s) of the reaction was(ere) still not found.

However, several well-established characteristics of the reaction between GSH and hemin may now be explained. The entire structure of hemin was altered by GSH (Figure 4, Figure 5 and Figure 6), indicating the decline in its spectrum (Figure 3A) [25,26,27,28,65]. The binding of GSH to hemin is thought to reduce the hydrophobicity of hemin [26]. GSH–hemin adducts and their hydroxy derivatives were less hydrophobic than hemin (Figure 4A). During the course of the reaction, the iron of hemin is released [28,31], relieving the cytotoxic potential of hemin. Free iron is less dangerous for cells than the iron entrapped in hemin. Iron binds to hemin through coordination bonds with nitrogen, which bonding is weakened in hydroxy–GSH–hemin adducts (Figure 6C). Hydroxy–GSH–hemin adducts easily release iron in the presence of reducing agents, such as GSH itself and/or ascorbic acid [31].

An intriguing feature of the proposed novel chemical mechanism is the lack of stereo specificity, as multiple isomers of GSH–hemin adducts were formed (Figure 4A,C and Figure 5). From a chemical point of view, the reaction of hemin with GSH is interesting, because the protoporphyrin ring of hemin is deteriorated by the direct opening of the ethene bridges by HO-1 and different micromolecules (such as H_2_O_2_) [66]. On the other hand, hemin binds to GSH-S transferase [67]. The isoform S-transferase p exists in erythrocytes that serves the removal of circulating xenobiotics [68]. Future studies will shed more light on the reaction’s details, as well as whether or not the reaction is facilitated by GSTs.

### 4.3. GSH–Hematin Adducts Were Identified as Novel Metabolites in Experimental Hemolysates

Next, two different sets of RBCs’ extracts were prepared. The first extract was prepared immediately after the isolation of RBCs from whole blood, as a picture of the content of RBCs at the time of isolation. In this extract, hematin was detected, while GSH–hematin adducts were found in traces at the exact *m*/*z* (461.12) of GSH–hemin adducts (Figure 8A). This observation was anticipated, because heme is bound covalently to the tertiary structure of hemoglobin and is unable to react with GSH. The autoxidation of hemoglobin has recently been found to yield a small amount of free heme inside healthy RBCs [50]. This amount of heme can contribute to the traces of GSH–hematin detected here. The latter extract of RBCs prepared at a time (1 h) after their hypotonic lysis and left to allow denaturation of hemoblobin, leading to heme oxidation and release in the RBC hemolysate. GSH–hematin adducts, as well as their hydroxy derivatives, were identified in abundance in this case (Figure 7A and Figure 8A). MS fragmentation data, complementary to *m*/*z*, supported our identification of these molecular ions (Figure 7C and Figure 8B). Their identification provides experimental evidence of the inter-reaction of hematin with GSH, both derived from RBCs. This inter-reaction proceeded via the proposed nucleophilic addition mechanism and led to the disintegration of hematin.

Hemolysis in vivo, in different pathological conditions, is followed by the accelerated oxidation of nearby amino acids in hemoglobin and heme loss in the presence of H_2_O_2_ and other oxidants [10]. In our experimental hemolysate, where GSH–hematin adducts were identified, acetonitrile was added to accelerate hematin release [51]. This study adds more information to previous pioneering studies that indicated that GSH is being a trap for free heme in the cytosol of RBCs [65]. Furthermore, GSH was proposed to defend the membrane of RBCs from injury by free heme [27]. However, recent studies have shown that partially oxygenated hemoglobin has an increased affinity with cellular membranes, which the antioxidant system of RBCs is, possibly, not fully able to reach [69]. Nevertheless, the affinity of hemin in the presence of GSH is reduced for both cytoskeletal proteins and the membrane lipid core, as shown by the subcellular fractionation of RBCs [27]. Moreover, the incubation of intact erythrocytes with hemin is followed by a time-dependent decrease in membrane-associated hemin [28].

On the other hand, it is interesting to note that hemin itself (at >5 μM for at least 1 h) induces hemolysis of intact RBCs [28]. Mechanistically, hemin provokes eryptosis [70], impairing the ability of RBCs to keep cation gradients [25]; by interacting with spectrin [71], it destabilizes the cytoskeleton of RBCs [72]. As found here, when hemin is added extracellularly in RBCs’ suspension at 20 μΜ for 30 min, GSH–hemin adducts were detected, as was the case for molecular ions as GSH–hematin adducts (Figure 7A). The unavoidable conclusion is that GSH, despite its abundance in RBCs (1 mM to 2 mM), cannot rescue them from hemin-induced hemolysis. Different parameters can contribute to this, as the mechanisms of hemin-induced hemolysis *per se*, as well as the rate of hemin import into RBCs [73]. Based on this observation, it can be concluded that the whole quantity of heme in each RBC (around 19.7 mM heme is packed in hemoglobin) is not able to be scavenged by the formation of GSH–hematin adducts. We believe that the mechanism of GSH–hematin adducts formation could be a homeostatic mechanism to react rapidly and in situ with heme that are leaked early by hemoblobin. In this way, RBCs would be helped in maintaining their redox balance and avoiding the self-propagating, free heme-induced hemolysis. In the more severe damage to RBCs, multiple homeostatic mechanisms exist to counterbalance the effects (as discussed in the Introduction).

### 4.4. Future Studies Regarding the Possible Biological Significance of GSH–Hematin Adducts Formation

A group of anemias associated with severe jaundice, due to genetic disorders of GSH metabolism, summarized in diseases database, such as KEGG (entry number H00668), depicts the crucial role of GSH in the homeostasis of RBCs. GSH–hematin adducts, as conditional formed metabolites, could be part of the human metabolome, as in aged RBCs [74]. In addition, hemoglobinopathic RBCs easily loose heme through hemichromes. Sickle cell disease RBCs are characterized by a high GSH turnover, as proven by decreased GSH content and elevation in its precursors [75], in association with elevated levels of non-heme iron [28,76]. Future studies will decipher whether GSH–hematin adducts and their derivatives are indeed novel metabolites of diagnostic value in disorders such as hemoglobinopathies. Furthermore, inter-individual variations in GSH levels of RBCs (0.4 mM to 3.0 mM) [17] suggest that the GSH level in each person may impact the formation of GSH–hemin/hematin adducts. The preventive effect of GSH against free heme cytotoxicity may be of potential clinical value in the treatment of hemolytic syndromes, brain hemorrhages (stroke), and other disorders, characterized by the release of vast quantities of heme and subsequent cell death. The crucial interplay of hematin and GSH needs to further explored, in light of the recently discovered GSH–hematin adducts and their derivatives.

## 5. Conclusions

In the present study, the chemical mechanism of reaction between GSH and hemin/hematin was delineated, yielding to the formation of newly presented chemical compounds. This reaction leads to the structural deterioration of hemin, thereby indicating the prominent protective action of GSH in hemin-induced cytotoxicity. Moreover, the identification of GSH–hematin adducts and their derivatives in extracts of human hemolysates implies that they are important metabolites in RBCs’ homeostasis.

## Data Availability

All of the data is contained within the article and the Supplementary Materials.

## Supplementary Materials

The following supporting information can be downloaded at: https://www.mdpi.com/article/10.3390/antiox11101959/s1, Figure S1: Detection of hemin and hematin as 616.18 *m*/*z* ion. (**A**,**B**). K562 cells were treated with 50 μΜ hemin in presence and absence of NAC (2 mM) for 24 h. Extraction of the LMW metabolites (5 × 10^6^ cells) and LC-MS/MS analysis took place, as described in the Materials and Methods section. An ion chromatograph of the 616.18 *m*/*z* (M-Cl) + (C_34_H_32_ClFeΝ_4_O_4_) (M, hemin) is shown. (**C**,**D**) RBCs (~2 × 10^9^) were subjected to the same extraction protocol, immediately after their isolation or incubated with 20 μΜ hemin (RPMI, 37 °C, 30 min). An ion chromatograph of the 616.18 *m*/*z* (M'-OH) + (C_34_H_32_OHFeΝ_4_O_4_) (M', hematin) is shown. The area under (AA) the curve of hemin is shown; compared with K562 cells treated with both NAC and hemin, the area of hemin-only treated cells is less, as found recently by direct measurements of hemin intracellular content (pyridine hemochromagen assay) [31]. Hemin-treated RBCs yielded a more abundant peak at 616.18 *m*/*z*, compared with untreated RBCs. A possible explanation for the partially diffused LC ion chromatograph peaks is the high quantity of the molecular ions. The slightly different RTs between hemin and/or hematin between the four samples was indicated, because each sample was run separately.
