# Supplementary material for: Glutathione–Hemin/Hematin Adduct Formation to Disintegrate Cytotoxic Oxidant Hemin/Hematin in Human K562 Cells and Red Blood Cells’ Hemolysates: Impact of Glutathione on the Hemolytic Disorders and Homeostasis
Source: Antioxidants (Basel). 2022 Sep 30;11(10):1959. doi: 10.3390/antiox11101959 (PMC9598195; doi:10.3390/antiox11101959)
Supplement: Supplementary file 1 [file antioxidants-11-01959-s001.zip › antioxidants-1908976-supplementary.pdf]

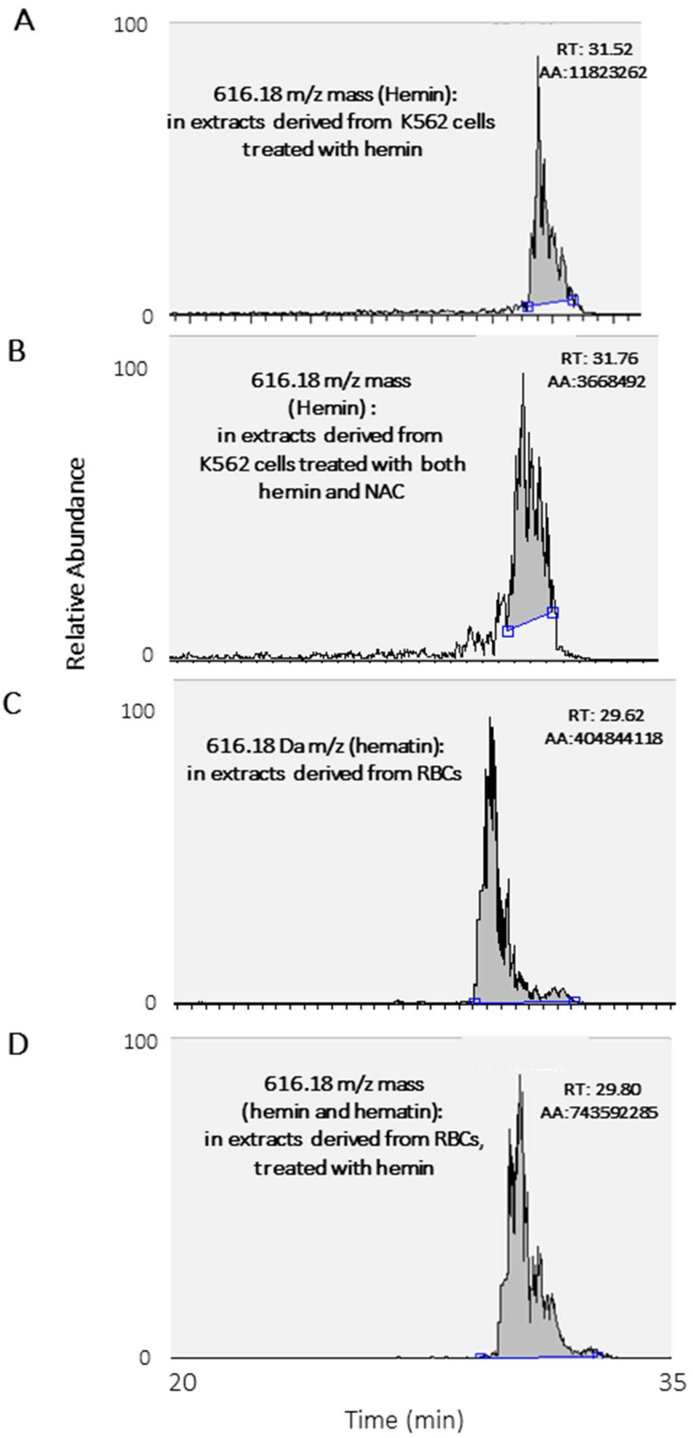

Figure S1. Detection of hemin and hematin as 616.18 m/z ion. (**A-B**). K562 cells were treated with 50μM hemin in presence and absence of NAC (2mM) for 24 h. Extraction of the LMW metabolites (5 x 10^6 cells) and LC-MS/MS analysis took place, as described in Materials and Methods. Ion chromatograph of the 616.18 m/z (M -Cl)+ ($C_{34}H_{32}ClFeN_4O_4$) (M, hemin) is shown. (**C-D**) RBCs (~2 x 10^9) were subjected to the same extraction protocol, immediately after their isolation or incubated with 20 μM hemin (RPMI, 37^{0}C, 30 min). Ion chromatograph of the 616.18 m/z (M΄-OH)+ ($C_{34}H_{32}OHFeN_4O_4$) (M΄, hematin) is shown. The area under (AA) the curve of hemin is shown and compared between K562 cells-treated with both NAC and hemin and hemin-only treated cells is less, as found recently by direct measurements of hemin intracellular content (pyridine hemochromagen asaay) [31].

Hemin-treated RBCs yielded a more abundant peak at 616.18 m/z, compared with untreated RBCs. A possible explanation for the partially diffused LC ion chromatograph peaks is the high quantity of the molecular ions. The slightly different RTs between hemin and/or hematin between the four samples is justified, since each sample was run separately.
